# High impact of COVID-19 outbreak in a nursing home in the Nouvelle-Aquitaine region, France, March to April 2020

**DOI:** 10.1186/s12879-021-05890-6

**Published:** 2021-02-22

**Authors:** A. Bernadou, S. Bouges, M. Catroux, J. C. Rigaux, C. Laland, N. Levêque, U. Noury, S. Larrieu, S. Acef, D. Habold, F. Cazenave-Roblot, L. Filleul

**Affiliations:** 1 Sante publique France en région Nouvelle-Aquitaine, Bordeaux, France; 2 Agence régionale de Santé Nouvelle-Aquitaine, Délégation départementale de la Vienne, Poitiers, France; 3 Centre Hospitalier Universitaire de Poitiers, Poitiers, France; 4 La Puye, France; 5 Centre d’appui pour la Prévention des Infections Associées aux Soins de Nouvelle-Aquitaine, Poitiers, France; 6 Agence régionale de Santé Nouvelle-Aquitaine, Bordeaux, France

**Keywords:** COVID-19, Nursing home, Outbreak, France

## Abstract

**Background:**

Elderly people in nursing homes are particularly vulnerable to COVID-19 due to their age, the presence of comorbidities, and community living. On March 14, 2020, at the beginning of the first epidemic wave of COVID-19 in France, a cluster was reported in a nursing home in the Nouvelle-Aquitaine region. We monitored the outbreak as well as the infection prevention and control (IPC) measures implemented.

**Methods:**

A confirmed case was defined as laboratory-confirmed COVID-19 in a resident or staff member present in the nursing home between March 7 and May 1, 2020; and a probable case as a person presenting an acute respiratory illness after contact with a confirmed case. Symptomatic inpatient residents and symptomatic staff members were systematically tested for SARS-CoV-2. In addition, two screening sessions were held on site.

**Results:**

We identified 109 cases (98 confirmed, 11 probable). The attack rate was 66% among residents and 45% among staff. Half of all cases were identified during the screening sessions. One-quarter of cases had minor symptoms or were asymptomatic. The case fatality rate among residents was 29%. IPC measures were rapidly implemented such as the quarantine of residents, the reinforcement of staff personal protective equipment, and home quarantine of staff testing positive, which were supplemented in April by systematic controls at the entrance of the nursing home and the creation of additional staff break rooms.

**Conclusions:**

This outbreak confirmed the considerable health impact of SARS-CoV-2 transmission in a nursing home. In addition to the implementation of IPC measures, the early detection of cases through the screening of residents and staff is essential to identify asymptomatic and pre-symptomatic cases and limit the spread of the virus.

## Background

In Europe, the first reported case of SARS-CoV-2 was identified in the Nouvelle-Aquitaine region on January 24, 2020 [1]. Despite active contact tracing, no secondary case was detected. In the following weeks, the virus spread throughout the country, reaching 22,302 cases in France on March 24, including 789 in Nouvelle-Aquitaine [2].

On March 14, 2020, the University Hospital Centre (CHU) in Poitiers reported a confirmed COVID-19 case in a nursing home resident who was subsequently hospitalized. The coordinating physician of the nursing home promptly identified three other residents with suspected symptoms. They were hospitalized and biologically confirmed as SARS-CoV-2 positive on March 15. In this study, we monitored the epidemic dynamic in this nursing home and the infection prevention and control (IPC) measures implemented throughout this outbreak.

## Methods

### Nursing home description

On March 14, 2020, 88 residents were living in the nursing home and 104 professionals worked on site, including health care and non-health care staff, private medical practitioners, and kitchen staff. The main building consisted of four areas, including a protected living unit (specializing in the care of people suffering from Alzheimer’s or related diseases). Residents of the first three areas usually ate together in two dining rooms, with the staff eating in a separate common room. Nuns (estimated to number around 15) also lived in the nursing home but in a separate area of the building on their own.

### Case definitions

A confirmed case was defined as a person present in the nursing home with a positive SARS-CoV-2 RT-PCR on a respiratory sample between March 7 and May 1, 2020.

A probable case was defined as a person present in the nursing home who had acute respiratory illness (ARI) within 14 days after being in contact with a confirmed case but who had not tested positive for SARS-CoV-2 within 21 days after the onset of symptoms.

In the absence of effective protective measures, a contact was defined as a person who shared the same living area as a confirmed case, had direct contact with a case (face-to-face, within 1 m, for any length of time), had given or received acts of hygiene or care with a confirmed case, had shared an enclosed space with a case for at least 15 consecutive minutes or cumulated over 24 h, or had remained face-to-face with a case during several episodes of coughing or sneezing.

### Testing strategy

Symptomatic residents hospitalized in Poitiers CHU were systematically and immediately tested for SARS-CoV-2. Symptomatic staff members were directed to be tested in Poitiers CHU or a private laboratory for the duration of the outbreak. In addition, two screening sessions were performed by a mobile team from Poitiers CHU that collected respiratory samples on site. The virology laboratory of Poitiers CHU carried out SARS-CoV2 RT-PCR tests. During the first screening session on March 18, symptomatic staff members were invited to be tested; whereas during the second screening session on April 7 and 10, all staff members and residents who had never tested positive for SARS-CoV-2 were tested, regardless of the presence of symptoms.

### Follow-up of COVID-19 residents

COVID-19 residents were examined by nurses twice a day for the monitoring of vital signs (temperature, blood pressure, and oxygen saturation). A physician was present in the facility on a daily basis and consulted patients at least twice a week or more if necessary. The primary criterion for transferring a resident to hospital was respiratory distress associated with the benefit/risk of hospitalization for the patient. This decision was subject to the agreement of the patient’s family.

### Data collection

The coordinating physician and staff of the nursing home completed a standardized table throughout the outbreak in order to collect information on demographic characteristics, room location, clinical symptoms, and biological results for each case. Symptoms were categorized as major (fever, cough, dyspnea, ageusia/agnosia) or minor (myalgia, asthenia, headache, digestive disorder, rhinitis, sore throat). The Regional Office of the French Public Health Agency (Santé publique France) provided data on the contact tracing of biologically confirmed staff members, and Poitiers CHU provided data on hospitalized residents and screening results. With regard to the community of nuns, no investigation was possible.

### Statistical analysis

We calculated attack rates (COVID-19 cases compared to the study population) as well as case fatality rates (people who died from COVID-19 compared to cases) for both residents and staff as well as for total cases (confirmed plus probable) and confirmed cases alone. We compared the characteristics of hospitalized cases and deceased cases. All comparisons of categorical variables were performed with Chi2 or Fisher’s test for low numbers. The comparison of means was performed with Student’s test. Analyses were performed with R software.

## Results

From March 7 to May 1, 2020, we identified 109 cases, involving 98 confirmed and 11 probable cases, in the nursing home in Nouvelle-Aquitaine. Among these cases, 58 were residents (47 confirmed, 11 probable), 47 staff members (all confirmed), and 4 nuns (all confirmed). The overall attack rate was 55% (66% among residents, 45% among staff), and for confirmed cases, 51% (53% among residents, 45% among staff).

The two screening sessions resulted in the detection of 57 cases. At the first screening, 11/23 staff members were diagnosed (i.e., positivity rate of 48%). At the second screening, 32/64 residents and 14/63 staff members were diagnosed (i.e., positivity rates of 50 and 23%, respectively).

**Fig. 1:**
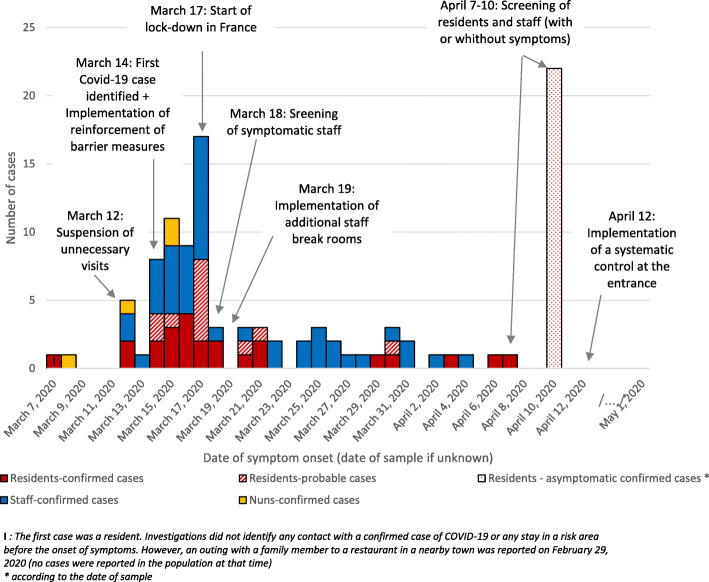
COVID-19 cases according to the date of onset (*N* = 106) and the control measures implemented in the nursing home, Nouvelle-Aquitaine, from March 7 to May 1, 2020

The first case was identified on March 14, 2020, 7 days after the onset of first symptoms. The epidemic curve showed the simultaneous circulation of the virus among residents and staff (Fig. 1) with a peak on March 17. In the next 3 weeks, new cases occurred almost every day until the implementation of the last screening.

Among the 47 staff members meeting the confirmed or probable case definition, 44 were interviewed (93%).

Patient characteristics are described in Table 1. Most cases were female (sex ratio = 0.12), with a mean age of 88 years among residents and 41 years among staff members. The majority of cases (73%) experienced major symptoms (including 36 residents and 41 staff members), 3 had minor symptoms (all staff members), and 22 were asymptomatic (all residents). Among the symptomatic residents, almost all of them had at least one risk factor other than age (mostly cardiac disease and high blood pressure), while among staff members, a minority (14%) reported at least one risk factor.

**Table 1:** Characteristics of COVID-19 cases among residents and staff in a nursing home, Nouvelle-Aquitaine, from March 7 to May 1, 2020

	Residents (*N* = 58)		Staff (*N* = 47)	
	*N*	%	N	%
**Type of cases**				
Confirmed	47	81%	47	100%
Probable	11	19%	0	0%
**Mean age (interquartile range)**	88	(87–93)	41	(30–53)
**Gender**				
Male	10	17%	1	2%
Female	48	83%	46	98%
**Hospitalization**				
Yes	16	28%	0	0%
No	42	72%	47	100%
**Death**				
Yes	17	29%	0	0%
No	41	71%	47	100%
**Risk factors (other than age)**^**a,b**^				
Hypertension	21	58%	2	4%
Diabetes	3	8%	1	2%
Obesity	8	22%	2	4%
Respiratory disease	9	25%	0	0%
Cardiac disease	24	67%	0	0%
Neurovascular disease	11	31%	0	0%
Renal disease	3	8%	1	2%
Immunodeficiency	3	8%	1	2%
**Symptoms**^**a**^				
None	22	38%	0	0%
Minor only	0	0%	3	7%
Major	36	62%	41	93%
*Types of symptoms*				
Fever^c^	28	48%	29	62%
Cough	17	29%	24	51%
Digestive problems^d^	3	5%	13	28%
Dyspnea	NA		7	15%
Asthenia	NA		36	77%
Myalgia	NA		29	62%
Headache	NA		32	68%
Anosmia/Ageusia	NA		14	30%

*NA* Not available; ^a^ among interviewed staff only (*n* = 44);^b^ among residents with symptoms (*n* = 36); ^c^ objectified fever or feeling of fever; ^d^ diarrhea, vomiting, nausea

Overall, 16 residents were hospitalized, and 17 residents died (case fatality rate 29% overall in total and 19% among confirmed cases), including 6 died in the hospital. Hospitalized and deceased residents were not different from other resident cases in terms of age, sex, and risk factors.

Among staff, all professional groups were affected (nurses, nursing assistants, other health care workers, service staff, kitchen staff, and administrative staff), although the highest attack rate was observed among service staff (56%), nurses (54%), and nursing assistants (50%).

Among the 33 staff members interviewed about their on-site presence before the onset of symptoms (75%), 29 reported working within 2 days before symptom onset, 24 on the same day, and 23 within 4 days afterwards. All were put on sick leave on the day on which they tested positive or before.

All areas of the nursing home were affected, and no significant difference was observed in the distribution of symptomatic and asymptomatic cases by room location.

### Infection prevention and control (IPC) measures

For all residents with ARI, droplet measures (quarantine in rooms, masks, and hydroalcoholic solutions at the entrance of the room) were systematically implemented [3]. On March 12, 2020, as a preventive measure in the context of the COVID-19 epidemic, all visits were suspended (Fig. 1). On March 14, 2020, additional IPC measures were implemented, such as active case surveillance (including testing of symptomatic staff), quarantine of all residents, reinforcement of staff personal protective equipment, and home quarantine of staff testing positive. On March 18, a mobile team from Poitiers CHU performed the first screening session, reminded staff members about IPC measures, provided instructions on face mask use, and supplied staff with protective equipment (face masks, gowns, gloves, etc.). They also proposed making organizational changes such as the creation of additional staff break rooms in order to respect physical distancing during mealtimes. Following the persistence of cases until April, a second screening session was organized; and from April 12, controls at the nursing home entrance were implemented, including the signing of a register, distribution of protective equipment, and measurement of temperatures (systematic from April 20).

## Discussion

Nursing homes accommodate elderly people who often suffer from significant comorbidities that make them, in addition to their age, highly vulnerable to COVID-19. This outbreak confirmed the considerable health impact of SARS-CoV-2 transmission in a long-term care facility, with a very high attack rate (66%) and severity (29% case fatality rate) among residents. Comparable but lower attack and case fatality rates were obtained when considering confirmed cases alone (52 and 19%, respectively). These results are consistent with previous studies that showed among nursing home residents a prevalence of COVID-19 of 40–64% in the UK and USA and COVID-19-associated mortality of 17–34% in UK, USA, Spain, and Germany [4–8]. Our study also showed that staff may be widely contaminated and thus contribute to the spread of the virus within the facility [9].

The late identification of the index case (7 days after onset) contributed to the circulation and rapid explosion of the virus among both residents and staff. Indeed, reducing the delay between symptom onset and case isolation has been shown to be effective in reducing the spread of emerging infectious diseases [10, 11]. In the present outbreak, many cases might have been infected within a relatively short time period because of the delayed identification, and therefore isolation, of the first case. Furthermore, at the beginning of the outbreak, there was a delayed identification and home quarantine of symptomatic staff who experienced early minor symptoms and remained at work while waiting for the test results at the beginning of the outbreak. One recommendation that should be implemented is the systematic home quarantine of all staff in nursing homes as soon as symptoms appear, even minor ones. However, this recommendation can be difficult to implement, because nursing homes often face a staff shortage, making the situation worse. Indeed, a study in the USA highlighted that nursing homes “having any residents or staff with Covid-19 were significantly more likely to experience shortages of all types of staff” [12].

Furthermore, although IPC measures were implemented early on, staff members at the beginning of the outbreak continued to share their meals in a single enclosed break room without social distancing measures. This might have strongly contributed to the viral circulation among staff members [13, 14] and then throughout the entire nursing home. Indeed, cases were observed among staff in all jobs, even those without any contact with residents.

After the peak on March 17, new cases persisted despite the implementation of additional IPC measures. Not all residents and staff could be tested during the first screening session due to the national strategy targeting only symptomatic individuals, partly due to the low testing capacity at the national level. Extended early screening, regardless of symptoms, could have allowed for the early identification of asymptomatic or pre-symptomatic cases [15]. Indeed, investigations have shown a significant proportion of infections in asymptomatic residents (38%), which is consistent with a study that showed that half of all residents in a long-term care facility who tested positive for COVID-19 were asymptomatic or pre-symptomatic [8, 16, 17]. The presence of asymptomatic or pre-symptomatic individuals is an important factor of virus transmission [9, 18], and therefore their rapid identification is crucial in order to isolate them and implement early strict IPC measures [19].

As the initial screening did not target asymptomatic residents and staff, this may have led to the non-detection of some asymptomatic cases. We may therefore have underestimated the attack rates and overestimated the case fatality rate in this cluster. However, our results are consistent with those of similar studies [4–6, 8].

From this outbreak, several observations were made and actions implemented:

- The need for an alert system, especially for long-term care facilities, to rapidly identify any symptomatic residents or staff to ensure the implementation of IPC measures without waiting for confirmation: this system was set up at the national level on March 28, 2020 [20];
- A broad testing strategy of exposed staff and residents, regardless of symptoms;
- Suspension of unnecessary social visits;
- Organizational changes with systematic controls at the entrance of the nursing home, one-way passageways, and dedicated sectors grouping the identified COVID-19 cases (including dedicated staff).

This outbreak confirmed the extreme fragility of nursing homes in the face of the COVID pandemic, and more widely, infectious respiratory diseases due to the extremely fragile population, limited human resources, and difficulty in implementing IPC measures. In the context of the current second wave of COVID-19, but also to prevent the outbreak of other respiratory viruses such as influenza, the preparation and protection of long-term care facilities should be reconsidered and reinforced for the future.

## Data Availability

The datasets generated and/or analyzed during the current study are not publicly available due to participants’ privacy but are available from the corresponding author on request.

## Abbreviations

ARI: Acute respiratory illness
IPC: Infection prevention and control
